# The impact of perception on institutional delivery service utilization in Northwest Ethiopia: the health belief model

**DOI:** 10.1186/s12884-022-05140-w

**Published:** 2022-11-06

**Authors:** Adane Nigusie, Telake Azale, Mezgebu Yitayal, Lemma Derseh

**Affiliations:** 1 Department of Health Promotion and Health Behavior, Institute of Public Health, College of Medicine and Health Sciences, University of Gondar, Gondar, Ethiopia; 2 Departemenr of Health Systems and Policy, Institute of Public Health, College of Medicine and Health Sciences, University of Gondar, Gondar, Ethiopia; 3 Departement of Epidemiology and Biostatics, Institute of Public Health, College of Medicine and Health Sciences, University of Gondar, Gondar, Ethiopia

**Keywords:** Health belief model, Perceived susceptibility, Cues to action, Health behavior

## Abstract

**Background:**

Ethiopia has been striving to promote institutional delivery through community wide programs. However, home is still the preferred place of delivery for most women encouraged by the community`s perception that delivery is a normal process and home is the ideal environment. The proportion of women using institutional delivery service is below the expected level. Therefore, we examined the impact of perception on institutional delivery service use by using the health belief model.

**Methods:**

A community-based cross-sectional study was conducted among 1,394 women who gave birth during the past 1 year from September to December 2019. A multistage sampling technique was used to select the study participants. Data were collected by using health belief model constructs, and structured and pretested questionnaire. Binary logistic regression was performed to identify factors associated with the outcome variable at 95% confidence level.

**Results:**

Institutional delivery service was used by 58.17% (95% CI: 55.57- 60.77%) of women. The study showed that high perceived susceptibility (AOR = 1.87; 95% CI 1.19–2.92), high cues to action (AOR = 1.57; 95% CI: 1.04–2.36), husbands with primary school education (AOR = 1.43; 95% CI 1.06–1.94), multiparty(5 or more) (AOR = 2.96; 95% CI 1.85–4.72), discussion on institutional delivery at home (AOR = 4.25; 95% CI 2.85–6.35), no close follow-up by health workers (AOR = 0.59;95% CI 0.39–0.88), regular antenatal care follow-up (AOR = 1.77;95% CI 1.23,2.58), health professionals lack of respect to clients (AOR = 2.32; 95% CI 1.45–3.79), and lack of health workers (AOR = 0.43;95% CI 0.29–0.61) were significantly associated with the utilization health behavior of institutional delivery service.

**Conclusion:**

The prevalence of institutional delivery in the study area was low. The current study revealed that among the health belief model construct perceived susceptibility and cues to action were significantly associated with the utilization behavior of institutional delivery service. On top of that strong follow-up of the community and home based discussion was a significant factor for the utilization behavior of institutional delivery service.

**Supplementary Information:**

The online version contains supplementary material available at 10.1186/s12884-022-05140-w.

## Background

Worldwide, the major causes of maternal deaths are due to direct obstetric complications including obstructed labor [1]. In 2014, births assisted by skilled health personnel increased to 71% from 59% to 1990 [2]. A care that is given for women during pregnancy, delivery and after delivery is vital for the well-being of both the mother and the new born [3].

Encouraging skilled delivery has a significant effect in reducing maternal morbidity and mortality; which has been practiced for more than two decades in Latin America and showed a visible progress. Pregnancy-related complications were high in Asia and Africa; and leading the world at large [4]. Maternal mortality in remote-rural communities who have poor access to health care remains high compared to urban setting [5]. For the improvement of maternal and new born child care in low and middle income countries, ensuring and promoting access to the ante-natal care and skilled birth attendant during delivery is a key strategy [6].

In Ethiopia, in spite of all the efforts made to improve the outcomes of maternal health, the number of women who get skilled birth attendant is still very low [7]. Direct obstetric complications are the major causes of maternal death in Ethiopia including prolonged/obstructed labor [8]. According to the 2015 united Nation (UN) and the 2019 Ethiopian Ministry of Health estimate, there is a significant progress on reducing the maternal mortality in Ethiopia, there is a significant progress on reducing the maternal mortality in Ethiopia [9]. The utilization of maternal health service particularly skilled birth attendant at health facility in Ethiopia increased substantially after 2005; from 6% in the 2000 and 2005 Ethiopia Demographic Health Survey (EDHS) to 10% in 2011 EDHS, 26% in 2016 EDHS [10] and reached 50% in 2019 [11], but still it is low as compared to the Health Sector Transformation Plan of Ethiopia (HSTP-II) [12].

Different studies conducted on institutional delivery service utilization identified individual level factors like perception, attitude and beliefs affecting health facility delivery service utilization [13–18].The determinants of individual’s health related behaviors and the way of motivating favorable changes can easily facilitated by behavioral theories and models, including the health belief model (HBM). The HBM was developed initially in the 1950s by social psychologists in the U.S. Public Health Service to explain the widespread failure of people to participate in programs to prevent and detect disease [19–22] Later, the model was extended to study people’s responses to symptoms [23] and their behaviors in response to a diagnosed illness, particularly adherence to medical regimens [24] and modified by Rostenstock in 1990 [25] as one of the value expectancy theory, develop preventive and utilization health behavior theories.

The HBM assumes that the likelihood of behavior (delivery at health facility) is predicted by (1) the individual’s perceived severity of giving birth out of health facility, (2) the individual’s perceived susceptibility of birth complications due to giving birth out of health facility, (3) the perceived net benefit of institutional delivery (if the perceived benefit outweighs the perceived barrier), (4) perceived barrier to institutional delivery, (5) the individual’s self-efficacy for institutional delivery service utilization, and (6) exposure to cues-to-action (information that motivates for institutional delivery service use) [26, 27].

Many of the previous study conducted in Ethiopia on institutional delivery service use and factors associated with it did not use the HBM constructs to understand the relationship between perceptions and institutional delivery service use with the help of skilled health personnel. Therefore, the current study aimed to determine the magnitude of institutional delivery service use and its associated factors in rural Central Gondar Zone, North West Ethiopia using the HBM.

## Methods

### Study design, period and setting

A community based cross sectional study was conducted among women in 15 rural kebele (the smallest administrative units of Ethiopia) of Central Gondar zone, Northwest Ethiopia, from September to December, 2019. Central Gondar zone is found in Amhara National Regional State (ANRS) and its capital city, Gondar is located 727Kms away from Addis Ababa, the capital city of Ethiopia. According to the 2007 census, the Zone has a population of 2,288,442 inhabitants of whom 462,952 were women of reproductive age. According to the 2019 Central Gondar zone health department report, there were 14 districts (2 urban and 12 rural), 75 health centers and 9 hospitals in the zone [28].

### Sample size

We calculated the sample size using single population proportion formula;

- n = z (a/2)^2^p (1-p)/d^2^
- n = Sample size
- Z_a/2_= 1.96 standard score corresponding to 95% CI
- d = 0.03 margin of error
- p = 0.271 (Proportion of institutional delivery service utilization in Amhara region) [10].
- d = 0.05
- n = z _(a/2)_^2^p (1-p)/d^2^
- n = (1.96)^2^0.271(1-0.271)/ (0.03)^2^= **845**
- n = 845, since there is design effect we multiply by 1.5 then it will be 1,267 with 10% non-response rate, it will give 1,394.

Therefore the maximum sample size was 1,394 women who gave birth in the last 12 months.

### Sampling procedures

We employed a multistage sampling technique to select the study participants. By taking 20–30% as a rule of thumb for representativeness, in the first stage among 12 rural districts of the zone we selected randomly two sample rural districts (Dembiya and Wogera) which are composed of 51 kebeles (24 kebele from Dembiya and 27 kebele from Wogera). In the second stage, among the 51 kebeles 15 kebeles were selected with a simple random sampling technique from the two selected districts proportionally. Within the selected kebele, households having the eligible study participants (women who gave birth within the past 1 year) were identified by using the maternal and child registration book of HEWs. Within the registration book, women who gave birth within the past 1 year in each kebele were identified before the survey; and we used this as a sampling frame to select the study participants. The HEWs are health cadres who are high school graduates and received 1-year training to deliver packages of preventive and health promotion services and few basic curative services.

Then, systematic random sampling was used within k^th^ (5th ) cases from each kebele to get representative participants until we addressed the required samples. Proportional sampling was done based on the number of women who gave birth in the last year living in the selected kebele using last year’s pregnant women’s registration book as sampling frame in the health post. In connection to this, we selected randomly the first study participant from the list.

### Data collection tool, procedure, and quality control

Appropriate questions of the health belief model were designed by reviewing theoretical aspects of the model, articles, and accessible guides for each of the HBM constructs using a 5-point Likert scale, ranging from “strongly disagree” to “strongly agree” (score range of 1–5) [25, 26, 29–32]. In addition, a structured questionnaire which was developed by reviewing literatures [33–40] was developed for the socio-demographic and institutional delivery service. The questionnaire was piloted (field tested) to make the questionnaire clear. It was initially prepared in English and translated to the local language Amharic and back translated to English by language experts to see the consistency.

Based on the six HBM construct 37 questions were included for the health belief model construct, i.e. five questions of “perceived susceptibility” with a score range of 5–25, seven questions of “perceived severity” with a score range of 7–35, six questions of “perceived benefits” with score range of 6–30, eight questions of “perceived barriers” with score range of 8–40, three questions of “self-efficacy” with a score range of 3–15, and eight questions of “cues to action” with a score range of 8–40).

The entire questionnaire was pretested on 70 women (5% of the entire sample size) in the rural communities of West Gojjam zone, Bahir Dar Zuria districts which has similar context with the study area.

The main survey data were collected by ten nurses and supervised by three public health officers. Two days training was given for the data collectors and supervisors about the objectives and data collection process by the principal investigator. The data were checked for accuracy and consistency daily.

To minimize potential bias, we have conducted different strategies at the design, data collection and analysis stage. The sample size was sufficiently large to estimate the prevalence with adequate precision. The study population was clearly stated at the design stage, trying to achieving high response rate (≥ 80%) to prevent non-response bias, this was facilitated by using questionnaires that are not too long and don’t take too much time to complete. Field-testing / piloting of questionnaire was conducted in order to improve and refine it, necessary training was given for the data collectors and supervisors which helps to prevent information and interviewer bias. To avoid response bias for the likert types of questions we have carefully designing our survey questionnaire by involving different experts for ensuring the content validity, we have provided a simple, exhaustive set of answer options, we also use precise and simple language, it was structured appropriately, we have trying to personalize the survey by keeping our target audience in mind, and continuously track the metrics to be measured. To avoid confounding bias, we have used multivariable analysis during the analysis stage.

### Study variables

The outcome variable of the study was institutional delivery service use which was defined or coded as “Yes” if women reported that they gave their most recent birth (within the last 1 year) at health institution, and “No” if otherwise.

The independent variables of the study includes basic socio-demographic information (age, marital status, educational status of mothers, educational status of the husband, family size and parity or number of child birth), health message delivery system and supportive supervision ( home based discussion on institutional delivery service use, information delivery system, follow-up by health workers, history of health facility on institutional delivery service given and respectful health care delivery practice), maternal health service use behavior and characteristics of the health facility (ANC follow-up, time and cost invested for the service, place of birth, status of child birth, location of health facility and health workers) and the HBM constructs (perceived susceptibility, perceived benefits, perceived barriers, self-efficacy and cues to action).

### Data processing and analysis

The collected data were checked for completeness, consistency, and missing values; coded and entered, using Epi Data version 3.1 and cleaned and analyzed using STATA software version14.1.

Using descriptive methods, the data was summarized, prevalence of institutional delivery service use was determined and odds ratios (OR) were determined using logistic regression. The data obtained from individuals in each household were pooled to create a single large data set then the studies used the number of individuals institutional delivery service use analyzed as the statistical n value, which is we assume the data gathered at each kebele to be an independent measurement so that we can use simple logistic regression by ignoring clustering [41].

The HBM constructs originally comprised of an item with a five 5-point scale of question responses (1 = strongly disagree, 2 = disagree, 3 = neutral, 4 = agree, 5 = strongly agree) were computed in to another scale response category by collapsing responses for 1 and 2 into a disagree category and 4 and 5 into an agree category, yielding a 3-point scale: 1 = disagree, 2 = neutral, and 3 = agree. Then, dichotomized scale responses were generated by collapsing responses for 1 through 3 from the original scale to 0 = disagree and 4 and 5 to 1 = agree. The rationale of dichotomization between 3 and 4 from the original scale was that the preface of questions regards people who answered higher than or equal to 4 as those who agreed with the statement in an item [42]. Then, those respondents who scored above 60% of the total construct score were considered as high and the remaining were low. The cutoff point for this categorization was calculated using the demarcation threshold formula: cutoff point = (over all highest score-over all lowest score)/_2_+ total lowest score. In addition, the standardized mean of each construct was calculated by dividing the mean score by the number of questions in order to make the mean importance of each dimension of HBM constructs comparable.

The effect of different variables including the HBM constructs on institutional delivery service use was explored using crude and adjusted odds ratios. After checking the correlation of independent variables, significance was determined using crude and adjusted odds ratios with 95% confidence level. To determine the association between the different predictor variables with the dependent variable, first bi-variable analysis between each independent variable and outcome variable was investigated using a binary logistic regression model and then all variables having p-value < 0.2 in the bi-variable analysis were considered as a criterion for variable selection for inclusion into a multivariable model. So, all variables with a p-value of < 0.2 in the bi-variable were used for multi-variable logistic regression.

Adjusted Odds Ratios (AOR) with 95% Confidence Intervals (CI) was calculated to show the presence and strength of associations. A variable with p-value of less than 0.05 in the multivariable logistic regression model was considered as statistically significant.

## Results

### Socio-economic and demographic characteristics of the respondents

In this study a total of 1,389 women who gave birth in the last 12 months were interviewed with 99.6% response rate. The mean age of the respondents was 30 years old (SD = ± 0.15). In this study, 1,105 (79.55%) of the respondents could not read and write. Regarding their parity status, 644 (46.36%) had 3–4 child birth. Meanwhile 1,254 (90.2%) of the respondents were married (Table 1).

**Table 1:** Socio-economic and demographic characteristics of study participants in Central Gondar zone, Northwest Ethiopia, 2021

Variables		Number	Percent
Age	18–24	170	12.24
	25–34	855	61.56
	35 and above	364	26.21
Educational status of the respondents	Unable to read and write	1,105	79.55
	Elementary/Primary	210	15.12
	Secondary school and above	74	5.33
Marital status	Currently Married	1,254	90.28
	Currently not Married	135	9.72
Family size	Less than 5	532	38.30
	5 and above	857	61.70
Educational status of the husbands (n = 1,254)	Unable to read and write	767	55.22
	Elementary	469	33.77
	Secondary school and above	18	1.30
Parity/number of child birth	Less than or equal to two	309	22.25
	3–4	644	46.36
	Greater than or equal to 5	436	31.39

### Health message delivery and supportive-supervision system

Respondents were asked about the health message delivery and supportive supervision system undertaken in the local context. The study showed that more than two-third 969 (69.76%) of the respondents have a home based discussion on institutional delivery. With respect to health facility history, 833 (59.97%) of the respondents were reported that the health facility in child birth had bad history (Table 2).

**Table 2:** Health message delivery and supportive-supervision system in Central Gondar zone, Northwest Ethiopia, 2021

Variables		Number	Percent
Home based discussion on institutional delivery	Yes	969	69.76
	No	420	30.24
No close follow-up by health workers	Yes	1,129	81.28
	No	260	18.72
Poor health information delivery system	Yes	595	42.84
	No	794	57.16
Bad history of health facility in child birth	Yes	556	40.03
	No	833	59.97
Health professionals lack of respect to clients	Yes	181	13.08
	No	1,208	86.97

### Institutional delivery service use

The study indicated that two-third 943 (67.89%) of the respondents had regular ANC follow-up in their last pregnancy, and more than half 808 (58.17%) of the women delivered their last child at health institution. However, 543(39.09%) of the respondents reported that the service given at the health facilities were time-consuming and costly (Table 3).

**Table 3:** Maternal health service utilization history of women in Central Gondar zone, Northwest Ethiopia, 2021

Variable		Number	Percent
Regular ANC follow-up	Yes	943	67.89
	No	446	32.11
Service given in health facility is time-consuming and costly	Yes	543	39.09
	No	846	60.91
Place of birth for the last baby	Health facility	808	58.17
	Home	581	41.83
Status of last child birth	Planned	1,045	75.23
	Unplanned	344	24.77
Location of health facility is too far	Yes	628	45.21
	No	761	54.79
Lack of health workers	Yes	415	29.88
	No	974	78.12

### Health Belief Model (HBM) constructs

The standardized mean score of each dimension of HBM constructs was calculated by dividing the mean score by the number of questions. The “self-efficacy” had the highest mean (3.82), followed by “cues to action” (3.75), “perceived benefits” (3.73), and “perceived susceptibility” (3.10). Whereas the lowest mean belonged to “perceived barriers” (2.66) (Table 4).

**Table 4:** Frequency distribution of mean, standard deviation and standardized mean of health belief model constructs for utilization behaviors of institutional delivery in the participants at Central Gondar zone, Northwest Ethiopia, 2021

Variables	Number of questions	Range of scores for questions	Mean	Sd.	Standardized mean	Minimum	Maximum
Perceived susceptibility	5	1–5	15.48	5.21	3.10	5	25
Perceived severity	7	1–5	21.65	7.34	3.09	7	35
Perceived benefit	6	1–5	22.40	5.06	3.73	8	30
Perceived barrier	8	1–5	21.31	7.37	2.66	8	39
Self-efficacy	3	1–5	11.45	2.51	3.82	3	15
Cues to action	8	1–5	29.97	4.65	3.75	13	40

The study showed that 821 (59.11%) of the study participants had high perceived benefit of institutional delivery, of which 589 (71.7%) gave birth at health facility. Near to three-fourth 1,013 (72.93%) of study participants had high cues to action for institutional delivery service use, of which 672 (66.3%) gave birth at health facility. Regarding the perceived susceptibility, 710 (51.12%) of the participant had low perceived susceptibility of birth complications for delivery out of health facility, of which only 312 (43.9%) gave birth at health facility for their last birth. Similarly, 716 (51.55%) of the study participants had low perceived severity of bringing delivery out of health facility, of which only 321 (44.8%) gave birth at health facility for their last birth (Table 5).

**Table 5:** Health belief model constructs for women’s utilization behavior of institutional delivery in Central Gondar zone, Northwest Ethiopia, 2021

Constructs		Place of delivery		Total n (%)
		Health facility n (%)	Home n (%)	
Perceived susceptibility to birth complications if delivery takes place out of a health facility	Low	312 (43.9)	398 (56.1)	710 (51.12)
	High	496 (73)	183 (27)	679 (48.88)
Perceived severity of bringing delivery out of health facility	Low	321 (44.8)	395 (55.2)	716 (51.55)
	High	487 (72.4)	186 (27.6)	673 (48.45)
Perceived benefit of institutional delivery	Low	219 (38.6)	349 (61.4)	568 (40.89)
	High	589 (71.7)	232 (28.3)	821 (59.11)
Perceived barrier of institutional delivery	Low	538 (72.3)	206 (27.7)	744 (53.56)
	High	270 (41.9)	375 (58.1)	645 (46.44)
Self-efficacy for institutional delivery service use	Low	157 (40.1)	235 (59.9)	392 (28.22)
	High	651 (65.3)	346 (34.7)	997 (71.78)
Cues to action for institutional delivery service use	Low	136 (36.2)	240 (68.2)	376 (27.07)
	High	672 (66.3)	341 (33.7)	1013 (72.93)

### Health belief model constructs and other factors associated with institutional delivery service use

Using bivariate analysis the relationship between institutional delivery service use and predictor variables was first assessed. In the bivariate analysis, perceived susceptibility, perceived severity, perceived benefit, perceived barrier, self-efficacy, cues to action, age of respondents, educational status of husband, parity, home based discussion on institutional delivery, no close follow-up by health workers, regular ANC follow-up, location of health facility, status of birth, service at health facility is time consuming and costly, poor information delivery system, bad history of health facility in child birth, lack of respectful health care practice and lack of health workers were statistically significantly associated with institutional delivery service use (p < 0.05).

The independent effect of each predictor variable was examined by performing a multivariate logistic regression, and the result is presented in Table 6 below. Accordingly, among the health belief model constructs perceived susceptibility and cues to action were significantly associated with institutional delivery service use; Women with high perceived susceptibility of birth complications were near to two folds (AOR = 1.87; 95% CI: 1.19, 2.92) more likely to give birth at health facility compared to women with low perceived susceptibility of birth complications. Similarly, women with high cues to action for institutional delivery service use were more than one -and half folds more likely to give birth at a health facility (AOR = 1.57;95% CI: 1.04,2.36) than those women with low cues to action for institutional delivery service use.

Women who had discussion on institutional delivery at home were 4.25 times more likely to give birth at health facility as compared to their counterparts (AOR = 4.25; 95% CI 2.85,6.35).Women who attended regular ANC visit for their last pregnancy were one –and more than half (AOR = 1.77; 95% CI: 1.23, 2.58) folds to give birth at health facility. The odds of institutional delivery were more than eight-and half folds (AOR = 8.51;95% CI 5.89, 12.28) among women who had a family size of less than five as compared to those women who had a family size greater than or equal to five. Husband educational status was significantly associated with institutional delivery service use; those with primary educational status were one –and near to half fold to give birth at health facility (AOR = 1.43 ;95% CI 1.06, 1.94) than those educational status was unable to read and write.

The odds of giving birth at health facility was more than two folds (AOR = 2.32; 95% CI 1.45, 3.71) among women who reported that there was respectful health care delivery as compared to their counter parts.

However, the odds of giving birth at health facility was 41% (AOR = 0.59; 95% CI: 0.39, 0.88) less likely among women who reported that there was no close follow-up by health workers compared to women who reported that there was close follow-up by health workers. Similarly, women who reported that there was lack of health workers were 67% less likely to give birth at health facility compared to women who reported that there was no lack of health workers (Table 6).

**Table 6:** HBM constructs and other factors associated with institutional delivery service use in Central Gondar zone, Northwest Ethiopia, 2021

Constructs and Variable		Institutional delivery		COR(95%CI)	AOR(95%CI)	*P*-value
		**Yes**	**No**			
Perceived susceptibility	Low	312	398	1.00	1.00	
	High	496	183	3.45 (2.76–4.33)*	1.87 (1.19–2.92)*	0.006
Perceived severity	Low	321	395	1.00	1.00	
	High	487	186	3.22 (2.57–4.03)*	1.01 (0.64–1.58)	0.981
Perceived benefit	Low	219	347	1.00	1.00	
	High	589	232	4.05 (3.22–5.08)*	0.87 (0.44–1.65)	0.643
Perceived barrier	Low	538	206	3.62 (2.89–4.54)*	1.29 (0.78–2.13)	0.323
	High	270	375	1.00	1.00	
Self-efficacy	Low	157	235	1.00	1.00	
	High	651	346	2.82 (2.21–3.58)*	0.78 (0.56–1.25)	0.269
cues to action	Low	136	240	1.00	1.00	
	High	672	341	3.48 (2.72–4.45)*	1.57 (1.04–2.36)*	0.031
Age of the respondent	18–24	95	75	0.97 (0.69–1.35)	0.81 (0.51–1.23)	0.385
	25–34	484	371	1.00	1.00	
	≥ 35	229	135	1.30 (1.01, 1.67)*	1.26 (0.90–1.77)	0.174
Educational status of the respondent	Cannot read and write	600	505	1.00	1.00	
	Primary school	152	58	2.21 (1.59–3.05)*	1.21 (0.88–1.83)	0.363
	Secondary school and above	56	18	2.61 (1.52–4.51)*	1.22 (0.64–2.32)	0.543
Educational status of husband	Cannot read and write	349	418	1.00	1.00	
	Primary school	346	123	3.37 (2.62–4.32)*	1.43 (1.06–1.94)*	0.020
	Secondary school and above	12	6	2.40 (0.89–6.45)		
Parity/number of child birth	≤ 2	225	84	1.00	1.00	
	3–4	326	318	0.38 (0.29–0.51)*	2.78 (1.78–4.36)*	0.000
	≥ 5	257	179	0.54 (0.39–0.73)*	2.96 (1.85–4.72)*	0.000
Home based discussion on institutional delivery	Yes	715	254	9.90 (7.55–12.98)*	4.25 (2.85–6.35)*	0.000
	No	93	327	1.00	1.000	
No close follow-up by health workers	Yes	685	444	1.72 (1.31–2.25)*	0.59 (0.39–0.88)*	0.011
	No	123	137	1.00	1.00	
Regular ANC Follow up	Yes	677	266	6.12 (4.77–7.84)*	1.77 (1.23–2.58)*	0.003
	No	131	315	1.00	1.00	
Location of health facility is too far	Yes	246	382	0.23 (0.18–0.29)*	0.88 (0.62–1.25)	0.464
	No	562	199	1.00	1.00	
Status of birth	Planned	629	416	1.39 (1.09–1.78)*	1.22 (0.87–1.71)	0.257
	Unplanned	179	165	1.00	1.00	
Service given in health facility is time taken and costly	Yes	192	351	0.20 (0.16–0.26)*	0.69 (0.45–1.10)	0.122
	No	616	230	1.00	1.00	
Poor information delivery system	Yes	241	354	0.27 (0.22–0.34)*	1.18 (0.79–1.76)	0.411
	No	567	227	1.00	1.00	
Bad history of health facility in child birth	Yes	199	357	0.21 (0.16–0.26)*	0.69 (0.45–1.08)	0.106
	No	609	224	1.00	1.00	
Health professionals lack of respect to clients	Yes	136	45	2.41 (1.69–3.44)*	2.32 (1.45–3.79)*	0.000
	No	672	536	1.00	1.00	
Lack of health workers	Yes	142	273	0.24 (0.19–0.31)*	0.43 (0.29–0.61)*	0.000
	No	666	308	1.00	1.00	

* = *P*-value < 0.05

## Discussion

Health belief model is one of the most extensively used and robust individual level models to measure behaviors in high income settings. We used this model to assess institutional delivery service use in a rural setting and examine which constructs of the model best predict such a behavior.

The Ethiopian Federal Ministry of health has included and is implementing the health policy that provides free maternal health care services for all women during pregnancy, labor and post natal period in the governmental health facilities. However, the use of institutional delivery service among women was over half in the study area, Central Gondar zone, North West Ethiopia, which is not satisfactory. Among the health belief model constructs perceived susceptibility and cues to action were predictor variables for the use of institutional delivery. Other factors that were significantly associated with the use of institutional delivery service were educational status of husband, parity/number of child birth, home based discussion on institutional delivery, no close follow-up by health workers, regular ANC follow-up, health professionals lack of respect to clients and lack of health workers.

Utilization behavior of individual is driven by their perception to take over the action and the expectation of people to carry out their perception when the opportunity arises. Individual will be involved in the health behavior if and only if their perceived threat outweighs the barrier [26]. In this study, the use of institutional delivery was low as compared to other studies conducted in other parts of Ethiopia: Mana district [43], Pawe district [44], BenchMaji [45] and Boset [46]. This might be due to differences in socio-cultural context[47] the current study area was two different districts, low level of community readiness for the promotion of institutional delivery [48], poor awareness creation on the consequences and complications of birth outcome at home [13] and lack of the integration of conscious raising program on institutional delivery service use with the cultural and social system in the community [49, 50].

Women’s perceived susceptibility to birth-related complications was significantly associated with utilization of health facility for birth. Poor awareness of community regarding the possible birth complications had a great impact on the health facility use during delivery service by the community at large [13, 16, 51]. The level of perception of women about the risks of giving birth at home could be determined based on their previous experiences of complications of home delivery by themselves, family, neighbors and the community at large; which in-turn determines the subsequent delivery places [31, 52–54]. Consequently, women may prefer giving birth at health facility based on their high perceived susceptibility to birth complications at home [55–57]. This implies that intensifying women’s perception regarding their susceptibility for birth complications at home over health facility could inspire them to visit health facility for every birth.

In the current study, cues to action for using health facility during child birth were positively associated with utilization health behavior for institutional delivery service.

Earlier studies showed that cues to action such as health learning materials like leaflet, poster, radio, newspaper; health workers respectful health care delivery, recommendations from religious fathers and kebele leaders [32, 58, 59], are important motivational factors to be involved in the desired behavior. Cues to action are motivational factors making women to be aware of the benefit of institutional delivery over home delivery and being alert whenever they think of giving childbirth [60–62].This implies that provision of different reminders by using locally available health learning material in order to address the community at large via considering the accessibility and availability of health learning material for the local context shall to be considered.

In this study, parity and home based discussion on institutional delivery service use were found to be significantly associated with health facility delivery care service use among study participants. This finding was in line with a study in Nepal [63] and in different areas of Ethiopia [64, 65]. Having home based discussion on different health issues has been also assessed in other studies [3] and indicates that it was a significant factor for the service use. Home based discussion on institutional delivery service use could enhance the uptake of the maternal health service in the large communities [66]. This implies that encouraging the community with a strong follow-up at a house hold level and making them to have a detailed discussion and taking appropriate solutions on barriers that hinder health care service uptake is a vital; and crucial strategy that everyone could takeover.

The study showed that, health professional’s lack of respect to clients and lack of health workers at health facility were significantly associated with the use of health facility for child birth among women with less than 1 year children in the study area. The practice of respectful health care delivery has high probability of positive impact on the service utilizer to visit again and build up a positive image by the large community [67, 68]. A health facility with low number of health care providers could have a negative impact on the service utilization; limited number of health care provender might not be serve the community as per the professional standard due to tiredness and fatigue, as a result they might not treat the clients respectfully [69–71]. This implies that the government, at each level of the health system tier, shall deploy a minimum number of health care providers as per the standards.

The odds of institutional delivery were higher among women who had a regular ANC follow-up in the current study area. This result was in-line with studies conducted in Mandura district, Pawi district, Hosanna, and rural area of Hadya zone in Ethiopia [72–75]. ANC follow-up provides opportunity for women to receive information about the benefits of institutional delivery.

Educational status of husband was found to be important determinants of institutional delivery service use. Women who had husbands with a primary school level education had a significant role in determining place of delivery. This finding was in agreement with other studies in Bangladesh, Sub-Saharan Africa, Eritrea and Nepal [76–80]. This might be due to the accessibility of the information and health education; service knowledge, and wise resource utilization that could be improved through educational level; and the preference of individuals for delivery at health facility would be improved easily by changing people’s attitude towards their preference of delivery [73, 81, 82]. This implies that strengthening adult education at the local level is a necessary and mandatory step for the improvement of institutional delivery service use.

The present study revealed that women who reported that there was no strong close follow-up by health workers were less likely to give birth in health facility compared to women who reported there was close follow-up by health workers. This finding is in line with an interventional study conducted in Melawi and Ethiopia in different area [83–85].The reason might be community who is closely advised and supervised by the health workers are often involved in, or consulted about, services, and service use; which makes them to be active utilizer of the services. This implies that there should be an established close follow-up system within the health facility and shall to be incorporated in the health workers performance appraisal systems.

### Strengths and limitations

Addressing the perception of rural women on the utilization health behavior of institutional delivery service using the constructs of health belief model could be the strengths of this study. The possible limitations of this study might be related to the focus of the health belief model i.e. the model emphasizes on individual level factors. Hence, determinants beyond the individual level factors like the social and structural factors may not have been addressed.

## Conclusion

The result of the current study indicated that perceived susceptibility, cues to action, parity, home based discussion on institutional delivery, close follow-up by health workers, respectful health care delivery practice, lack of health workers, husband educational status and regular ANC follow-up were some of the predictive variable which had the highest power in predicting the utilization behaviors of institutional delivery. Therefore, it is necessary to design an intervention to increase awareness in women and the community at large to promote utilization health behaviors; this can be addressed by strengthening peer-health education on the possible complications of birth at home and awareness creation activities using different social systems are recommended. Designing and establishing a platform for the follow-up of the community and a system of home based discussion enables the community to adopt the desirable behavior i.e. utilization behavior. Increasing the motivational factors i.e. cues to action and giving more attention for respectful health care delivery practice might also promote utilization of institutional delivery service.

## Supplementary Information

**Additional file 1.**

## Data Availability

The datasets generated and/or analyzed during the current study are available at University of Gondar, College of medicine and Health Science, Institute of Public Health in hard and soft copy repository [www.UoG.edu.et]. In addition the data are available from the authors upon reasonable request and with permission of the principal investigators (Adane Nigusie- E-mail adane_n@yahoo.com).

## Abbreviations

ANC: Ante Natal Care
ANRS: Amhara National Regional State
AOR: Adjusted Odds Ratios
BSC: Bachelor of Science
CI: Confidence Intervals
COR: Crude Odd Ratio
EDHS: Ethiopian Demographic Health Survey
HBM: Health Belief Model
HEW: Health Extension Workers
HSTP: Health Sector Transformation Plan
HW: Health Workers
UN: United Nation
